# Surveillance of Levofloxacin Resistance in *Helicobacter pylori* Isolates in Bogotá-Colombia (2009-2014)

**DOI:** 10.1371/journal.pone.0160007

**Published:** 2016-07-25

**Authors:** Alba A. Trespalacios-Rangél, William Otero, Azucena Arévalo-Galvis, Raúl A. Poutou-Piñales, Emiko Rimbara, David Y. Graham

**Affiliations:** 1 Grupo de Enfermedades Infecciosas, Departamento de Microbiología, Pontificia Universidad Javeriana, Bogotá, D.C, Colombia; 2 Unidad de Gastroenterología, Universidad Nacional de Colombia, Bogotá, D.C, Colombia; 3 Grupo de Biotecnología Ambiental e Industrial (GBAI). Departamento de Microbiología, Pontificia Universidad Javeriana, Bogotá, D.C, Colombia; 4 Department of Medicine, Michael E. DeBakey Veterans Affairs Medical Center, Houston TX, United States of America; 5 Baylor College of Medicine, Houston TX, United States of America; Second University of Naples, ITALY

## Abstract

Increased resistance of *Helicobacter pylori* to clarithromycin and metronidazole has resulted in recommendation to substitute fluoroquinolones for eradication therapy. The aims of the study were to determine the prevalence and changes in primary levofloxacin resistance related to *H*. *pylori gyr*A sequences. The study utilized *H*. *pylori* strains isolated from patients undergoing gastroscopy in Bogotá, Colombia from 2009 to 2014. Levofloxacin susceptibility was assessed by agar dilution. Mutations in *gyr*A sequences affecting the quinolone resistance-determining region (QRDR) were evaluated by direct sequencing. Overall, the mean prevalence of primary levofloxacin resistance was 18.2% (80 of 439 samples). Resistance increased from 11.8% (12/102) in 2009 to 27.3% (21/77) in 2014 (p = 0.001). *gyr*A mutations in levofloxacin resistant strains were present in QRDR positions 87 and 91. The most common mutation was N87I (43.8%, 35/80) followed by D91N (28.8%, 23/80) and N87K (11.3%, 9/80). Levofloxacin resistance increased markedly in Colombia during the six-year study period. Primary levofloxacin resistance was most often mediated by point mutations in *gyr*A, with N87I being the most common QRDR mutation related to levofloxacin resistance.

## Introduction

*Helicobacter pylori (H*. *pylori)* is an important human pathogen causing gastroduodenal inflammation that can result in duodenal ulcer, gastric ulcer, gastric adenocarcinoma and primary B-cell gastric lymphoma [1,2]. *H*. *pylori* has also been related to extragastric diseases, including iron deficiency anemia, idiopathic thrombocytopenic purpura and even colon adenocarcinoma [3]. The National Institutes of Health in the United States, the Maastricht Consensus conferences in Europe, Second Asia-Pacific Consensus Guidelines for *H*. *pylori* infection and the Canadian Consensus all recommended *H*. *pylori* eradication for the treatment/or prevention of these disorders in addition to reducing occurrence of new gastric cancers [4–9]. An increase *H*. *pylori* resistance to standard antibiotics such as clarithromycin, and metronidazole has been reported. Therefore, a reduction in triple therapy (proton pump inhibitors (PPI) plus clarithromycin and amoxicillin) treatment success has fallen below 80% for most countries. Escalating resistance to clarithromycin has urged the necessity to identify other treatment options [10–12]. Levofloxacin is a levorotary isomer of ofloxacin with known broad activity against Gram-negative and Gram-positive bacteria [13]. Levofloxacin mode of action is via bacterial type II topoisomerase inhibition, namely DNA gyrase and topoisomerase IV. The potential advantages of levofloxacin-containing triple therapy include the presence of an “*in vivo*” synergistic effect between quinolone antimicrobial agents and PPIs against *H*. *pylori* strains. However, quinolone resistance in *H*. *pylori* has been reported to be increasing and could undermine its efficacy [14–20].

The most common mechanism of fluoroquinolone resistance involves mutations in DNA gyrase and in DNA topoisomerase IV. These genes encode large enzymatic quaternary structures that consist of two pairs of subunits: GyrA and GyrB (DNA gyrase) and ParC and ParE (DNA topoisomerase IV). However, *H*. *pylori* lacks topoisomerase IV, thus fluoroquinolone resistance is likely due to mutations in the DNA gyrase gene (*gyr*A), encoding the DNA gyrase subunit A (GyrA) [14–19,21]. The absence of a secondary target for fluoroquinolones suggests a single modification in the *gyr*A gene could be sufficient to result in a fluoroquinolone-resistant phenotype. The amino acid substitutions observed in clinical strains have been primarily reported at positions 87 (Asn to Lys) and 91 (Asp to Gly, Asp to Asn, or Asp to Tyr) [22,23]. The presence of levofloxacin resistance in levofloxacin-containing therapy has a dramatic detrimental effect on treatment success [18]. Therefore, levofloxacin prescription therapy should be based on local resistance prevalence. The aims of the present study were to determine the rate of primary levofloxacin resistance in *H*. *pylori* (prevalence and changes) in patients from Bogotá, Colombia from 2009 to 2014. In addition, we determined *gyr*A sequence diversity of resistant strains.

## Results

### Patient Characteristics

**Fig 1:**
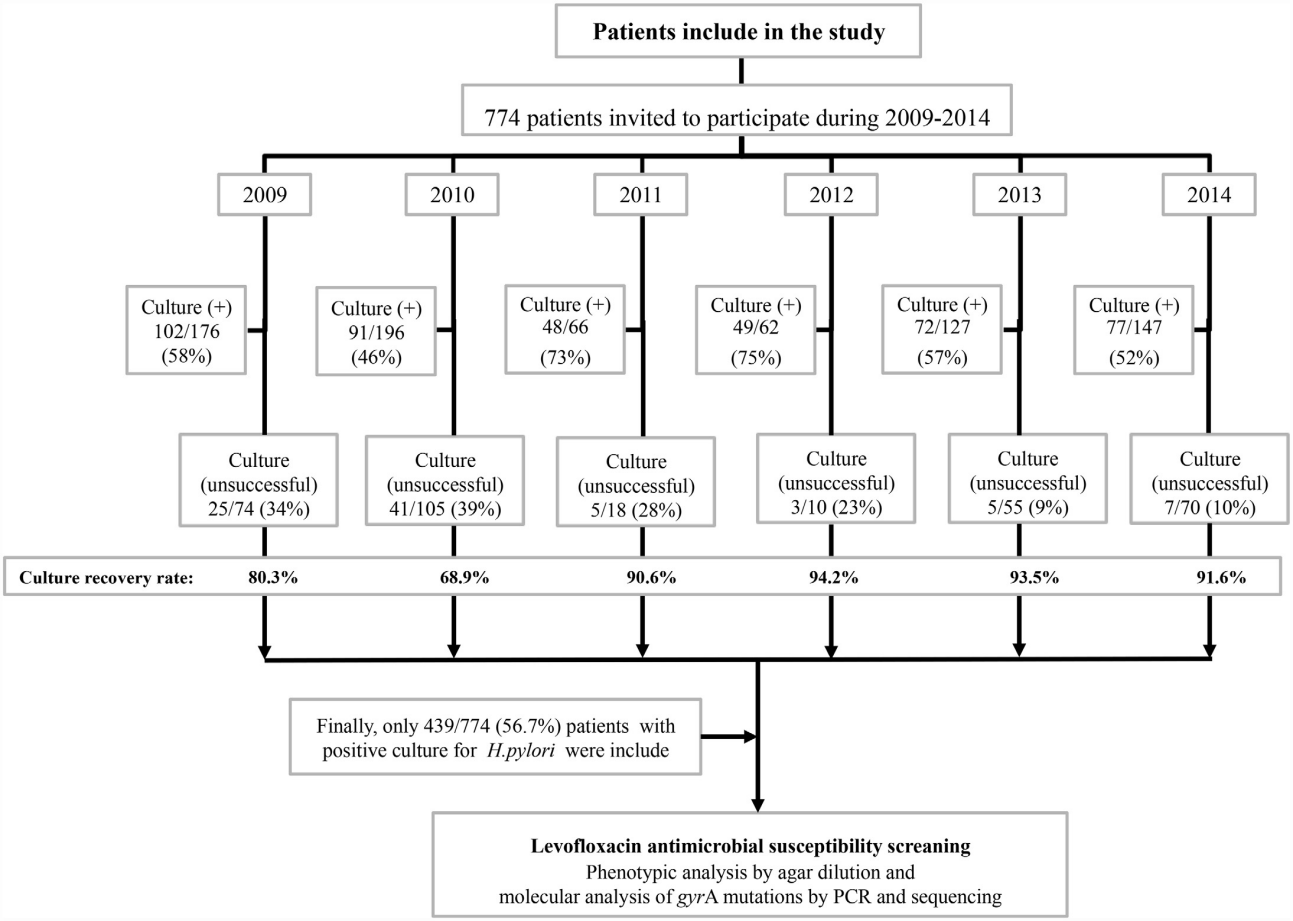
Patients included in the study. Diagram depicting details of number of patients/year and who was included in the study, based on *H*. *pylori* positive culture. Negative culture was related to *H*. *pylori* (negative), confirmed by histological analysis and rapid urease test or any other laboratory assay. Unsuccessful describes a sample where it was not possible to recover the isolate from a positive biopsy sample (positive for histological analysis and positive for rapid urease test and any other laboratory assay).

A total of 774 Colombian patients were enrolled from 2009 to 2014. After bacterial culture growth for *H*. *pylori*, only 439 (56.7%) patients were positive for *H*. *pylori* and were included in the study (Fig 1). Of the 439 *H*. *pylori* positive patients, 314/439 (71.6%) were women and 125/439 (38.4%) were men. Their ages ranged between 18 to 73 years old, with an average age of 46 ± 11 and 47 ± 13 for women and men, respectively. All of them were diagnosed with *H*. *pylori* chronic gastritis. All patients taking PPIs or H2-receptor antagonists were removed from this study, thus excluding patients with duodenal or gastric ulcers. The number of patients enrolled by year were: 2009: 102 subjects, 2010: 91 subjects, 2011: 48 subjects, 2012: 49 subjects, 2013: 72 subjects and 2014: 77 subjects.

### Prevalence of Levofloxacin Resistance

**Fig 2:**
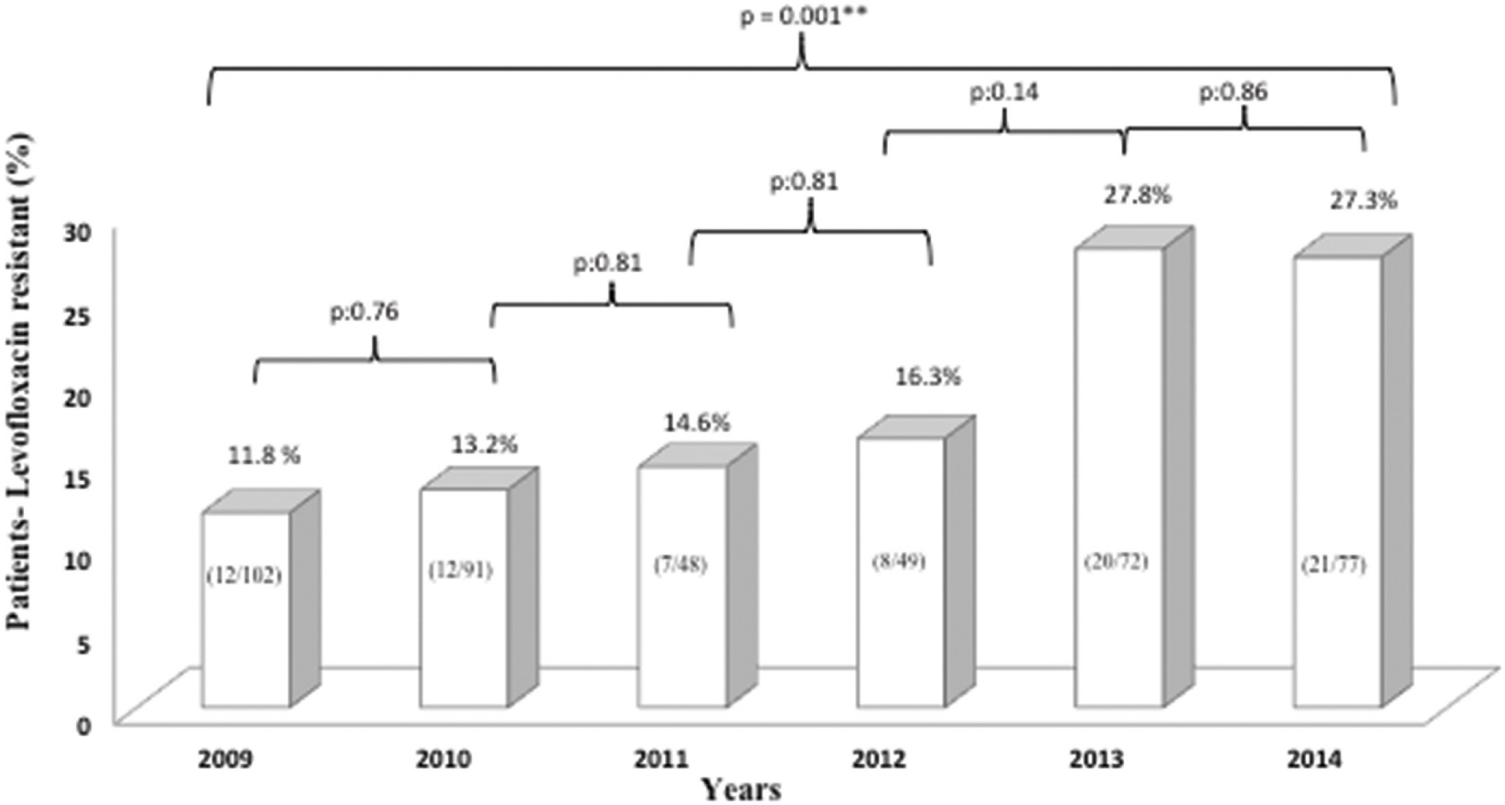
Yearly prevalence. Patients with *H*. *pylori* strains resistant to levofloxacin and statistical significance of differences in Bogotá-Colombia; between 2009 and 2014.

A total of 486 *H*. *pylori* isolates were obtained from 439 positive patients; meaning more than one isolate from the same patient, as has been previously reported [24–30]. The mean primary levofloxacin resistance rate was 18.2% (80/439). Minimum inhibitory concentrations (MICs) for levofloxacin resistant strains ranged from 1 to 64 μg/mL. The MICs distributions of susceptible strains ranged from 0.016 to 0.5 μg/mL. The levofloxacin resistance rate (per patient) studied for each year are in fig 2. Although resistance between each year was not significantly different, when comparing the resistant rate between 2009 and 2014 it was highly significant (p = 0.001) with a 15.5% difference (Fig 2).

### *gyr*A Mutations

Mutations in levofloxacin resistant strains were present in quinolone resistance-determining region (QRDR) at positions 87 and 91. The concordance score between phenotypic and genotypic test was 0.98. The most common resistant strain substitution was N87I, present in 43.8% (35/80), followed by D91N (28.8%; 23/80) and N87K present in 11.3% (9/80). Four of the patients with levofloxacin resistant strains (MICs from 1 to 8 μg/mL) did not present a *gyr*A mutation, and two patients with susceptible strains had N87I mutations. Levofloxacin MIC was 0.5 μg/mL for both N87I strains. Strains from two patients presented double mutations at 87 and 91 positions. Combination of double mutations at N87Y and D91G in one strain had a MIC of 1 μg/mL. The other double mutation (N87I and D91G) had a MIC of 32 μg/mL (Table 1).

**Table 1:** Association between minimal inhibitory concentrations levels (MICs) and *gyrA* nucleotides substitution and types of mutations in *gyr*A QRDR.

Patients with *gyr*A mutations in *H*. *pylori n* (%)	MIC (μg/mL)	*gyrA mutations data*		
		Nucleotide change	Aminoacid change	*gyr*A mutation and position
35/80 (43.8)	1–32	AAC → A**T**C	Asn **→ Ile**	N87I
9/80 (11.3)	2–16	AAC → AA**A**	Asn **→ Lys**	N87K
1/80 (1.3)	4	AAC → **TAT**	Asn **→ Tyr**	N87Y
23/80 (28.8)	2–32	GAT → G**G**T	Asp **→ Gly**	D91G
4/80 (5.0)	1	GAT → **A**AT	Asp **→ Asn**	D91N
1/80 (1.3)	2	GAT → **T**AT	Asp →**Tyr**	D91Y
1/80 (1.3)	1	AAC → **TAT** GAT → **A**AT	Asn **→ Tyr** Asp **→ Asn**	N87Y D91N
1/80 (1.3)	32	AAC → A**T**C GAT → **A**AT	Asn **→ Ile** Asp **→ Asn**	N87I D91G
4/80 (5)*	1–8	AAC GAT	Asn Asp	No Mutation
1/80 (1.3)*	0.5	AAC → A**T**C	Asn **→ Ile**	N87I

*conflicting results between genotypic and phenotypic resistant

## Discussion

Cammarota *et al*. proposed fluoroquinolones, such as levofloxacin as a first line of treatment as an alternative to clarithromycin against *H*. *pylori* [13]. In this work, we evaluated changes in the prevalence of “primary” levofloxacin resistance of *H*. *pylori* in isolates obtained in Bogotá, Colombia from January 2009 to August 2014. Distribution of patients per year is shown in fig 1, depicting the irregular nature of patient entry, since several other institutions performed endoscopies and did not participate in this study. Although the inclusion of patients was consecutive the referrals to our unit for endoscopy varied over time. In addition, the culture recovery rate for *H*. *pylori* from biopsy was more stable (90 to 94%) between 2011 and 2014 (Fig 1). Hence, prevalence of resistance to levofloxacin could be possibly higher than reported in this paper.

During the observation period, levofloxacin resistance significantly increased (p = 0.001) from 11.8% (12/102) in 2009 to 27.3% (21/77) in 2014 (Fig 2). Remarkably, only 14 years after it was introduced as an alternative treatment for *H*. *pylori* eradication, levofloxacin resistance increased to the point it was no longer an acceptable choice as an empiric therapy. Indeed, given that patients did not receive previous *H*. *pylori* treatment, most likely resistance could be the result of treatment for other infections such as respiratory infections, urinary tract infections, or diarrheal diseases. In addition, it is not possible to document fluoroquinolone consumption in Colombia (dose per 1,000 inhabitants/day) because it is possible to purchase this antibiotic without medical prescription; which allows for self-medication, favoring an increase in resistance. Camargo et al., (2014) study support this hypothesis, where *H*. *pylori* resistance to quinolones was reported to be approximately 15% in Latin America [31].

In Colombia the first line of treatment is triple therapy containing metronidazole, clarithromycin or levofloxacin in addition to amoxicillin and proton pump inhibitors for seven days. Various studies performed in Colombia since 1998 have evaluated *H*. *pylori* resistance to metronidazole. Since then, resistance to this antimicrobial agent has been reported to be greater than 80% [32]. In contrast, clarithromycin resistance has been reported to be low. A first study in 2007 by Alvarez et al., in the coffee growing region of the country reported a low resistance of 2.2% [33]. These results contrast with those from Bogota, the country’s capital where Trespalacios et al., (2013) reported a 13.6% resistance [34]. In addition, for the south of Colombia Acosta et al., (2014) reported a clarithromycin resistance of 4% [35]. Given these resistance variability’s for clarithromycin and few available studies for the different regions of the country, many gastroenterologists prefer to prescribe clarithromycin in regions where resistance to this antimicrobial is low. In contrast, for Bogota levofloxacin is commonly prescribed for first and second line therapies.

The scheme for second line of treatment mostly used is therapy with levofloxacin or quadruple therapies. However, a Colombian survey revealed physicians don’t follow these recommendations or international guidelines, but in an intuitive manner prescribe different regimens that for the most part have not been previously investigated [36]. However, this is the first study carried-out in Colombia to evaluate *H*. *pylori* resistance to levofloxacin in Bogota.

Other countries have also reported an increase in quinolone resistance. A systematic review published in 2010 indicated a global worldwide levofloxacin resistance of 16.2% (95% CI 14.4–18.0) with the highest level of resistance in Europe (24.1%); followed by a mid- resistance level in Asia (11.6%), with differences among Asian countries: Japan (14.9%), Taiwan 11.9% and Hong Kong 2.6% [15]. In addition, recent reports have suggested that prevalence of levofloxacin-resistant *H*. *pylori* strains is increasing. A recently steady annual increase has been observed, ranging from 18% up to >30% for Europe and Asia, Italy (18%), Turkey (23.8 to 29.5%), Vietnam (41.3%) and China (Beijing, 54.8%), [37–41]. Based on these findings it is advisable to restrict the empiric use of levofloxacin [42].

Quinolone resistance-determining region mutations in levofloxacin resistant *H*. *pylori* isolates had previously been reported. For strains isolated in our study we did not find any correlation between the mutation present and the level of resistance. However, higher levels of resistance were most likely in isolates with N87I and D91G mutations, i.e. with MIC values of 32 μg/mL. Our data agrees with reports from Asia and Europe [14–16]. Two of our susceptible strains had N87I mutations with MICs close to breakpoint (0.5 μg/mL). Conflicting results between genotypic and phenotypic resistance (Table 1) were found in those with *gyr*A wild-type sequences. 1/4 (25%), of resistant isolates without mutations in *gyr*A, had the mutation E463K in *gyr*B, hence this mutation is a candidate explaining isolate resistance. No mutations were identified in *gyr*B for the other 3/4 (75%) resistant isolates, without mutations in *gyr*A, (supplementary material). These data suggest the presence of additional resistant mechanisms involved in levofloxacin resistance [16,43]. According Rimbara *et al*., (2012) results, *H*. *pylori* isolates resistant to fluoroquinolones and without *gyr*A mutations are likely to have mutations in *gyr*B at positions 463 (supplementary material).

Discordant results were observed in four patients, where no phenotypic resistance was observed in the presence of *gyr*A sequence mutation (Table 1). These results may be possibly due to the levofloxacin resistance breakpoint used in this study (1 μg/mL). However, other studies describe isolates with *gyr*A mutations with a MIC of 0.5 μg/mL [44]. Additional studies are needed to correlate MIC and *gyrA* mutations, in order to obtain an accurate breakpoint for levofloxacin.

Overall, QRDR mutations were most often found at positions 87 and 91, and included N87I, N87K, N87Y, D91G, D91N and D91Y (Table 1). In 75/80 (94%) patients with resistant strains (MIC > 1 μg/mL), mutations occurred in the *gyr*A gene. This finding is consistent with mutations in *gyr*A occurrence, as a principal mechanism for levofloxacin resistance. As previously stated, four of our patients with resistant strains (MICs from 1 to 8 μg/mL) did not have a detectable mutation in QRDR of the *gyr*A gene. Thus, further studies are necessary to identify the resistance mechanism in those strains. As a case in point, Rimbara *et al*., (2011, 2012) reported *gyr*B mutations as a potential novel mechanism of resistance to fluoroquinolones in *H*. *pylori* [2,43]. However, other still unknown mechanisms could be also involved.

N87I mutation was detected in 43.8% (35/80) of resistant strains followed by D91G with a frequency of 28.8% (23/80) and N87K present in 11.3% (9/80), (Table 1). This distribution is different from that reported in Europe, Asia or Canada (North America), where the major mutation sites were N87K and D91G [14,45]. N87I mutation results from amino acid wild type *H*. *pylori* J99 substitution of aminoacid Thr87 to Ileu, with a C to T substitution at codon 87 ACC→ATC [21], (Table 1).

In conclusion, the prevalence of primary resistance to levofloxacin has been increasing in Colombia. Although, levofloxacin resistance was due to point mutation in *gyr*A, the mutations pattern differed from than reported in Europe. Results from this study evidenced the most common mutation in isolates resistant to levofloxacin was *gyr*A mutation N87I. This result contrasts with studies performed in France, where the most frequent mutation was N87K, followed by D91N, D91G and D91Y [46]. Mutation N87I has not been reported by the aforementioned studies. In a similar manner studies carried-out in Malaysia and Beijing report the most frequent *gyr*A mutations were: N87K, D91N, D91G, D91Y [16,47]. These results are different from those reported from Senegal, Africa; where the most common mutation was N87I followed by D91N. Low frequencies were reported for D91G and D91Y [45,48]. These last results agree with the ones reported in our study, where the most frequent mutation was N87I, followed by D91G and N87K. Therefore, it is important to continue performing studies that allow identifying *gyr*A mutations responsible for fluoroquinolone resistance and the geographical explanation for these differences. Surveillance of quinolone resistance in *H*. *pylori* should include assessment of new responsible mutations. The geographical area should be considered, to update the increasingly popular molecular tests.

## Materials and Methods

### Patients

Adult outpatients referred for gastroscopy at the Gastroenterology Unit of “*Clínica Fundadores”*, Bogotá D.C., Colombia, between January 2009 and August 2014, both positive for *H*. *pylori* rapid urease test (RUT) and histopathology (Giemsa stain) were enrolled. Written informed consent was obtained from each participating patient before enrolling the study. Research protocol was approved by the ethical committee of “*Pontificia Universidad Javeriana”* and “*Clínica Fundadores”* in Bogotá D.C., Colombia. Patients were excluded if they were taking PPI or H2-receptor antagonists four weeks prior to enrollment. Since the objective of the study was to assess primary resistance, those with previous anti-*H*. *pylori* treatment were also excluded, as were patients with concomitant illness. Biopsies from antrum and body of the stomach underwent bacterial culture and susceptibility testing and DNA extraction.

Bacterial strains, culture conditions, and determination of susceptibility to levofloxacin

Biopsies samples from antrum and body of the stomach were crushed in 0.5 mL PBS and cultured in Wilkins Chalgren Agar (Becton Dickinson, Heidelberg, Germany) containing 7% (v/v) horse blood, vancomycin (10 mg/L) and trimethoprim (5 mg/L). Petri dishes were incubated at 37°C under microaerophilic conditions for up to 14 days. *H*. *pylori* growth was confirmed by typical colony morphology, Gram stain, positive oxidase, catalase and urease tests.

Levofloxacin MICs for the recovered *H*. *pylori* strains were measured using the agar dilution method according to the guidelines of the National Committee for Clinical Laboratory Standards (CLSI), [49]. The resistance breakpoint was defined as ≥1 μg/mL [50,51]. The agar dilution method was performed by serial two fold dilution, mixing the antibiotic with molten Mueller-Hinton agar II, supplemented with 5% (v/v) sheep blood and 0.4% (v/v) Isovitalex (Becton Dickinson, MD) to reach the desired antibiotic concentration. 1 to 3 μL of a McFarland 2.0 adjusted inoculum were dropped on the surface of the Petri dishes; containing the solid media, followed by 35 ± 2°C for 72 h under microaerophilic conditions. For quality control *H*. *pylori* NCTC 11637, was used.

### DNA Extraction, PCR Amplification and Nucleotide Sequence Analysis

Genomic DNA of *H*. *pylori* strains was extracted using DNAzol^®^ kits (Invitrogen—USA). DNA was stored at -20°C until use. To detect gene mutations in the QRDRs subunit A of the DNA gyrase (*gyr*A), degenerated oligonucleotide primers reported by Tankovic *et al*., (2003); 5’-TTT RGC TTA TTC MAT GAG CGT-3’ and 5’-GCA GAC GGC TTG GTA RAA TA-3’ were used. Primers were synthesized by Invitrogen-USA. The size of the amplified fragments of *gyr*A was 428 bp. The PCR was performed with PCR Master Mix—Promega (WI, USA) using 1.0 μL DNA template, 1.0 pmol/L of each primer, in a final mix reaction volume of 50 μL. The cycling conditions were first 3 minutes at 94°C followed by 35 cycles of 1 min denaturation at 94°C, 1 min annealing at 57°C and 1 min extension at 72°C [52]. PCR products were analyzed by visualizing in 1.5% (w/v) agarose gel electrophoresis, followed by SYBR Green (Invitrogen-USA) staining for 30 minutes. PCR product purification and direct sequencing was performed by Macrogen, Korea. The oligonucleotides used for *gyr*A PCR were also used for DNA sequencing. The sequences were compared to *H*. *pylori gyr*A gene published sequence (GeneBank accession No. L29481). PCR was performed for all strains (susceptible and resistant) and direct sequencing was obtained for all PCR products.

### Data Analysis

Frequencies and percentages were used to describe *H*. *pylori* antimicrobial isolate resistance. Statistical significances for resistance rates among the different collection years were analyzed by the chi-squared test (χ^2^) with p < 0.05 considered as statistically significant. Phenotypic susceptibility was evaluated by MIC in μg/mL. Genotypic resistance was determined by the presence or lack of a mutation involved with resistance. Degree of agreement (concordance) between phenotypic and genotypic resistance was performed using Cohen’s kappa coefficient. All statistical analyses were performed using the statistical software package SPSS 18.0 for Windows (SPSS, Chicago, IL, USA).
